# N-Acetyl Cysteine Mitigates the Acute Effects of Cocaine-Induced Toxicity in Astroglia-Like Cells

**DOI:** 10.1371/journal.pone.0114285

**Published:** 2015-01-24

**Authors:** Ramesh B. Badisa, Sanjay S. Kumar, Elizabeth Mazzio, Rasheda D. Haughbrook, John R. Allen, Michael W. Davidson, Cheryl A. Fitch-Pye, Carl B. Goodman

**Affiliations:** 1 College of Pharmacy and Pharmaceutical Sciences, Florida A&M University, Tallahassee, Florida, United States of America; 2 Department of Biomedical Science, College of Medicine, Florida State University, Tallahassee, Florida, United States of America; 3 Department of Biology, Florida A&M University, Tallahassee, Florida, United States of America; 4 National High Magnetic Field Laboratory, Florida State University, Tallahassee, Florida, United States of America; 5 Department of Biological Science, Florida State University, Tallahassee, Florida, United States of America; University of Colorado, UNITED STATES

## Abstract

Cocaine has a short half-life of only about an hour but its effects, predominantly on the central nervous system (CNS), are fairly long-lasting. Of all cells within the CNS, astrocytes may be the first to display cocaine toxicity owing to their relative abundance in the brain. Cocaine entry could trigger several early response changes that adversely affect their survival, and inhibiting these changes could conversely increase their rate of survival. In order to identify these changes and the minimal concentrations of cocaine that can elicit them *in vitro*, rat *C6* astroglia-like cells were treated with cocaine (2–4 mM for 1h) and assayed for alterations in gross cell morphology, cytoplasmic vacuolation, viability, reactive oxygen species (ROS) generation, glutathione (GSH) levels, cell membrane integrity, F-actin cytoskeleton, and histone methylation. We report here that all of the above identified features are significantly altered by cocaine, and may collectively represent the key pathology underlying acute toxicity-mediated death of astroglia-like cells. Pretreatment of the cells with the clinically available antioxidant N-acetyl cysteine (NAC, 5 mM for 30 min) inhibited these changes during subsequent application of cocaine and mitigated cocaine-induced toxicity. Despite repeated cocaine exposure, NAC pretreated cells remained highly viable and post NAC treatment also increased viability of cocaine treated cells to a smaller yet significant level. We show further that this alleviation by NAC is mediated through an increase in GSH levels in the cells. These findings, coupled with the fact that astrocytes maintain neuronal integrity, suggest that compounds which target and mitigate these early toxic changes in astrocytes could have a potentially broad therapeutic role in cocaine-induced CNS damage.

## Introduction

Cocaine is an addictive and widely abused psychostimulant that can evade the protection of the blood brain barrier (BBB) to enter the brain and compromise its normal functioning. Cocaine's effects on biochemical processes in the CNS is an area of active research, and how these cocaine-induced changes impact neurons and astrocytes is not well understood. Although acute exposure to cocaine has been shown to alter gene expression [1], it is the changed cell biochemistry that appears to underlie many of the clinical symptoms. Identification of early biochemical symptoms such as vacuolation and changes in mitochondrial membrane potential may offer clues about underlying mechanisms and therapeutic avenues. While the long-term/chronic effects of cocaine, including post-translational modifications such as acetylation, methylation [2, 3], phosphorylation have been well established in the literature, early precipitating events that lead to these chronic changes following acute exposure are much less understood. Furthermore, cocaine's ability to interfere with normal signaling pathways in neurons [4] has narrowed the focus of research within CNS to neurons, despite evidence that astrocytes–cells that provide both physical and chemical support to neurons [5] and maintain the integrity of the BBB [6]–are also vulnerable. The present study is geared towards unraveling the acute morphological and epigenetic changes in astrocytes upon exposure to cocaine. Incorporating data from our previous studies that focused on the chronic effects of cocaine [7, 8] and considering that astrocytes outnumber neurons in most brain regions [9], we postulate that toxic effects of cocaine manifest in astrocytes prior to any neuronal damage. Cocaine's entry into the brain through the BBB, known for its astroglial interaction [10, 11], may also expose astrocytes to cocaine sooner and for longer periods than any other cell-type in the CNS thereby enhancing their vulnerability to cocaine-induced toxicity. Because neurons depend on astrocytes for survival [12, 13], loss of astrocytes due to cocaine toxicity could ultimately lead to loss of neurons / neuronal function [14]–a circumstance that could possibly be avoided in the initial stages of cocaine addiction by protecting astrocytes from the acute effects of cocaine-induced toxicity. This study tests the hypothesis that inhibition of the acute effects of cocaine in astrocytes increases their survival.

The objectives of the present study are to identify various early response changes associated with acute exposure of astroglia-like cells to physiologically-relevant doses of cocaine *in vitro*; to determine the minimal doses that compromise their viability; and to investigate the role of NAC in mitigating cocaine-induced toxicity in these cells and determining its mode of action. To this end, we used a CNS derived rat *C6* astroglia-like cell line (CCL-107) which is astrocytic in origin and unlike other CNS cell lines, exhibits a high degree of similarity with human astrocytes in its gene expression [15] and enzymes [16]. Studies have also shown that this cell line contains undifferentiated glial cells [17] that release glial cell line-derived neurotrophic factors similar to astrocytes [18]. Taken together, these properties demonstrate that *C6* cell cultures behave like an astroglia-like cell line. In the past, *C6* cells have also been used extensively for *in vitro* drug abuse research [7, 8, 19–22] and in the study of astrocytic function [23–30].

## Materials and Methods

### Chemicals

All chemicals used were of analytical grade. RPMI 1640, fetal bovine serum (FBS), penicillin/streptomycin sulfate, amphotericin B, phosphate-buffered saline (PBS) and L-glutamine were obtained from Media Tech (Herndon, VA, USA). Cocaine (Ecgonine methyl ester benzoate) hydrochloride, crystal violet, dichlorodihydrofluorescin diacetate dye (H_2_DCFDA), L-glutaraldehyde, 0.5 M EDTA (ethylene diamine tetraacetic acid) solution, 5,5-dithiobis-2-nitrobenzoic acid (DTNB), nicotinamide adenosine dinucleotide phosphate (NADPH), 5-sulfosalicylic acid, NAC and trypan blue were obtained from Sigma-Aldrich (St. Louis, MO, USA) and used according to various protocols.

### Cell Culture

The CNS derived rat *C6* astroglia-like cell line (CCL-107) was purchased from the American Type Culture Collection (Rockville, MD, USA) and maintained as a monolayer culture as described before [7].

### Immunocytochemistry of Glial Fibrillary Acidic Protein

We assayed for the presence of glial fibrillary acidic protein (GFAP), an important marker protein expressed abundantly only in astrocytes, in *C6* cells. Cells were cultured in 96-well plates (5x10^4^/well) over night following which they were fixed in 4% paraformaldehyde, permeabilized in 0.1% triton X 100 in PBS and incubated with rabbit-anti-rat GFAP 1° antibody (ab7260) (Abcam, Cambridge, MA) for 2 h at room temp. Samples were washed in PBS and subsequently incubated with goat anti-rabbit Alexa Fluor 488 conjugate for 2 h at room temp. These were counterstained for nuclei with propidium iodide (PI) and photomicrographed using an inverted microscope with a 40x objective, CCD camera. Data was acquired using ToupTek View (TouTek Photonics Co, Zhejiang, China).

### Treatment with Cocaine and NAC

Cocaine hydrochloride stock (1M) and working stock solutions (80–160 mM) were prepared in PBS as described previously [7]. Cytotoxic studies were performed in polystyrene, flat bottom 96-well (0.32 cm^2^) microtiter plates (BD Labware, NJ, USA). Cells were seeded at a starting density of 2x10^4^ cells per well in a total volume of 195 μl of complete RPMI 1640 growth medium with 10% FBS and allowed to adhere overnight in an incubator (37°C, 5% CO_2_). Cocaine was added to the medium under sterile conditions using minimal volumes of the working stock solutions (5 μl/well) to achieve final concentrations of 2, 3 and 4 mM without disrupting pH. Untreated cells received equal volumes of PBS (5 μl/well) and served as vehicle controls. Treated samples were interspersed with controls in different wells of the same 96-well microtiter culture plates. Treatment with cocaine was carried out in an incubator (37°C; 5% CO_2_) and lasted for 1h to mimic its biological half-life in the body [31, 32] and the fact that a single-dose of cocaine in addicts wears off after an hour for typical amounts and routes of intake (National Institute of Drug Abuse, Research Report Series, March 2010, Bethesda, Maryland, USA). In a subset of experiments, cells were pretreated with 5 mM NAC [33] for 30 min prior to cocaine exposure, while in another set of experiments, 5 mM NAC (30 min) was added to cells post 1h cocaine exposure. Cell viability and GSH levels were assayed at the end of an hour-long incubation using methods outlined below.

### Assessment of Morphology and Vacuolation

To evaluate gross morphological changes including vacuolation, cells were stained with crystal violet (0.1%) and observed under an inverted phase contrast IX-70 Olympus microscope (Ontario, NY, USA) with a 40x objective. Photomicrographs were taken using an ocular video-camera system (MD35 Electronic eyepiece, Zhejiang JinCheng Scientific & Technology Co., Ltd, HangZhou, China) running C-Imaging System Software (Compix Inc. Cranberry Township, PA, USA).

### Assessment of Cell Viability

Cell viability was assessed by the crystal violet dye uptake assay, described previously [34]. Briefly, at the end of the 1h treatment, 100 μl of 0.25% glutaraldehyde was added to each well and the cultures incubated for 30 min to fix cells to the polystyrene surface of culture plates. The plates were then gently washed three times and air-dried. Following staining with 0.1% aqueous crystal violet dye (15 min), the plates were washed and air dried again. The dye in each well was extracted with 100 μl of 50 mM sodium phosphate mono basic solution containing 50% ethyl alcohol. Optical density (OD) measurements of incorporated dye in viable cells were obtained at 540 nm using a microplate reader (Bio-Tek Instruments Inc, Wincoski, VT, USA). Cell viability was re-confirmed using 3-(4,5-dimethylthiazol-2-yl)-5(3-carboxymethonyphenol)-2-(4-sulfophenyl)-2H tetrazolium (MTS) Cell Titer 96 AQueous One Solution Reagent kit (Promega, Madison, WI, USA) and titer plates were read for dye color in a micro plate reader at 490 nm.

### Measurements of Intracellular ROS

Cocaine-induced ROS release was measured with H_2_DCFDA dye in 96-well plates. Prior to cocaine exposure, 10 μM of the dye was loaded into cells [35] for 30 min and washed. Cells were subsequently treated with phenol red free media before exposure to cocaine. Plates were read using a micro plate fluorometer model 7620, Version 5.02, Cambridge Technology, Inc., (Watertown, MA, USA) with the excitation and emission filters set at 485 and 530 nm, respectively.

### Assessment of Total Cellular GSH Levels

Total cellular GSH was assayed according to [36]. Briefly, following cocaine treatment, cells were fixed with 0.25% glutaraldehyde for 30 min followed by gentle washing and air drying. Cells were then deproteinized with 2% 5-sulfosalicylic acid (10 μl/well) for 30 min at 37°C followed by incubation with 90 μl of a reaction mixture containing (in mM): 0.416 sodium EDTA, 0.416 NADPH, 0.835 DTNB and 0.083 sodium phosphate buffer (pH 7.5) for 30 min at 37°C. Absorbance of color was measured on a micro plate reader at 412 nm.

### Plasma Membrane Integrity Assay

Cell membrane integrity following exposure to various concentrations of cocaine was determined by measuring lactose dehydrogenase (LDH) release with CytoTox 96 non-radioactive assay kit (Promega) as per kit instructions provided by the manufacturer. Color intensity was measured using a micro plate reader at 490 nm.

### Fluorescence Microscopy for Nuclear and F-Actin Staining

Briefly, following cocaine treatment, cells were fixed in 4% paraformaldehyde for 15 min and subsequently permeabilized in 0.25% triton X 100 in PBS for 15 min. Stock solutions for fluorescent dyes (Life Technologies, Carlsbad, CA, USA) were made in ethanol (5 mg/ml) and diluted in HBSS before being added to cells. Final concentrations for PI (excitation: 535 nm, emission 617 nm) and Alexa Fluor 488 Phalloidin (excitation: 488 nm; emission: 520 nm) were 5 μg/ml and 6.6 μM respectively. Images show Alexa Fluor 488 Phalloidin and PI nuclear counterstain to corroborate changes in cytoskeleton F-actin tertiary structure. Samples were analyzed photographically using XDY-1 inverted fluorescent microscope with a 40x objective, CCD camera and data acquired using ToupTek View (TouTek Photonics Co, Zhejiang, China). Fluorescence intensity was acquired using Image J software, National Institutes of Health (NIH), Bethesda, Maryland, USA.

### Histone H3-K27 Methylation Assay

Global histone H3-K27 methylation assay kit (Epigentek, Farmingdale, NY, USA) was used to measure H3-K27 methylation in the cells. Briefly, cells (starting density: 1.5x10^6^) were seeded in 100 mm sterile culture dishes per 10 ml of complete DMEM containing 5.5 mM glucose and incubated overnight. They were exposed to cocaine (1h) on the following day and harvested by trypsinization. Histone extraction and methylation detection were done as per the protocol supplied by the manufacturer (Epigentek). OD measurements were obtained from a micro plate reader at 450 nm. The amount of methylation (ng/mg protein) in H3 histone was determined by the following equation: (OD/slope) × 1000. Slope was determined from the standard curve of the positive control (H3-K27) supplied with the kit.

### Statistical Analysis

Experimental results are presented as the mean ± standard error of the mean (S.E.M.). The data were analyzed for significance by one-way ANOVA and then compared using Dunnett's multiple comparison tests in GraphPad Prism Software, version 5 (San Diego, CA, USA). The lethal concentration of cocaine (mM) needed to kill 50% of cells (LC_50_), was determined as described earlier [37].

## Results

### Detection of GFAP in *C6* Cells

**Figure 1:**
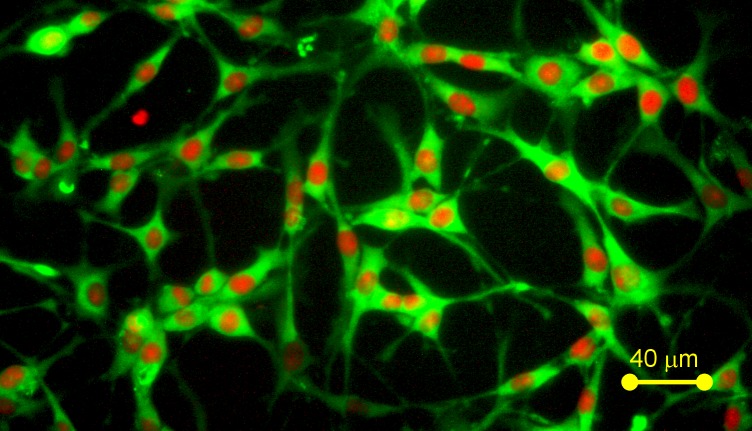
Detection of glial fibrillary acidic protein marker in *C6* astroglia-like cells. Cells were incubated with rabbit-anti-rat GFAP 1° antibody and then with goat anti-rabbit Alexa Fluor 488 conjugate for 2 h. Samples were counterstained for nuclei with PI and photomicrographed using an inverted microscope with a 40x objective.

The *C6* cells used in this study were initially derived from rat brain tumors [38] and shown to express GFAP [29], a characteristic marker protein of astrocytes. Thus, these cells, used extensively in drug abuse studies, can be as considered astrocytic in function. We reconfirmed the astrocytic nature of *C6* cells by assaying their GFAP expression. We found that C6 cells indeed expressed high levels of GFAP (Fig. 1).

### The Early Response Changes

Acute exposure of astroglia-like cells to cocaine (2–4 mM for 1h) elicited changes in morphological features, induction of vacuolation, ROS generation, alteration in GSH levels, cell membrane disruption, damage to cytoskeleton, and lysine methylation. These changes will be collectively dubbed as the “early response changes”, described below.

### Morphological Changes and Sensitivity to Cocaine Exposure

**Figure 2:**
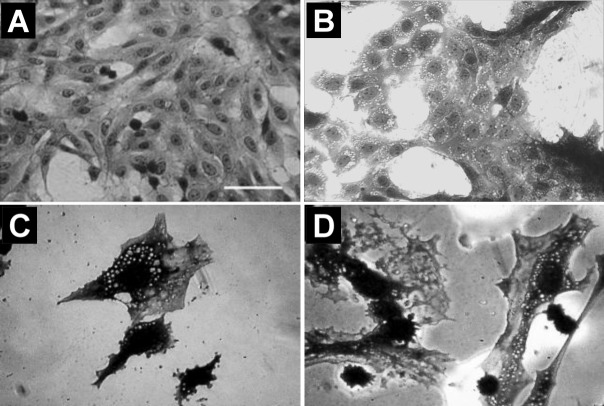
Gross morphological features of astroglia-like cells in culture during acute exposure to cocaine. Cells were treated with PBS (control) (**A**) and 2–4 mM cocaine (**B-D**) for 1h. Optical images are of crystal violet stained cells taken with an inverted phase contrast IX-70 Olympus microscope with a 40x objective. Note the conspicuous cocaine-induced vacuoles in the cytoplasm (**B**-**D**). Scale bar: 50 μm.

One of the first and foremost effects of toxic drug exposure is a change in cell morphology. Microscopic observation of crystal violet stained astroglia-like cells revealed that an hour-long exposure to cocaine was enough to evoke profound alterations in general architecture including cell-shape. The whole cell morphology became irregular, intercellular gaps expanded and there was a marked vacuolization as a function of cocaine concentration (Fig. 2B-D). Despite these changes, there was no apparent evidence of nuclear lysis at any of the cocaine doses used. As changes in cell morphology are indicative of cell toxicity, it is evident that astroglia-like cells are extremely sensitive to acute exposure of cocaine.

### Cell Viability

**Figure 3:**
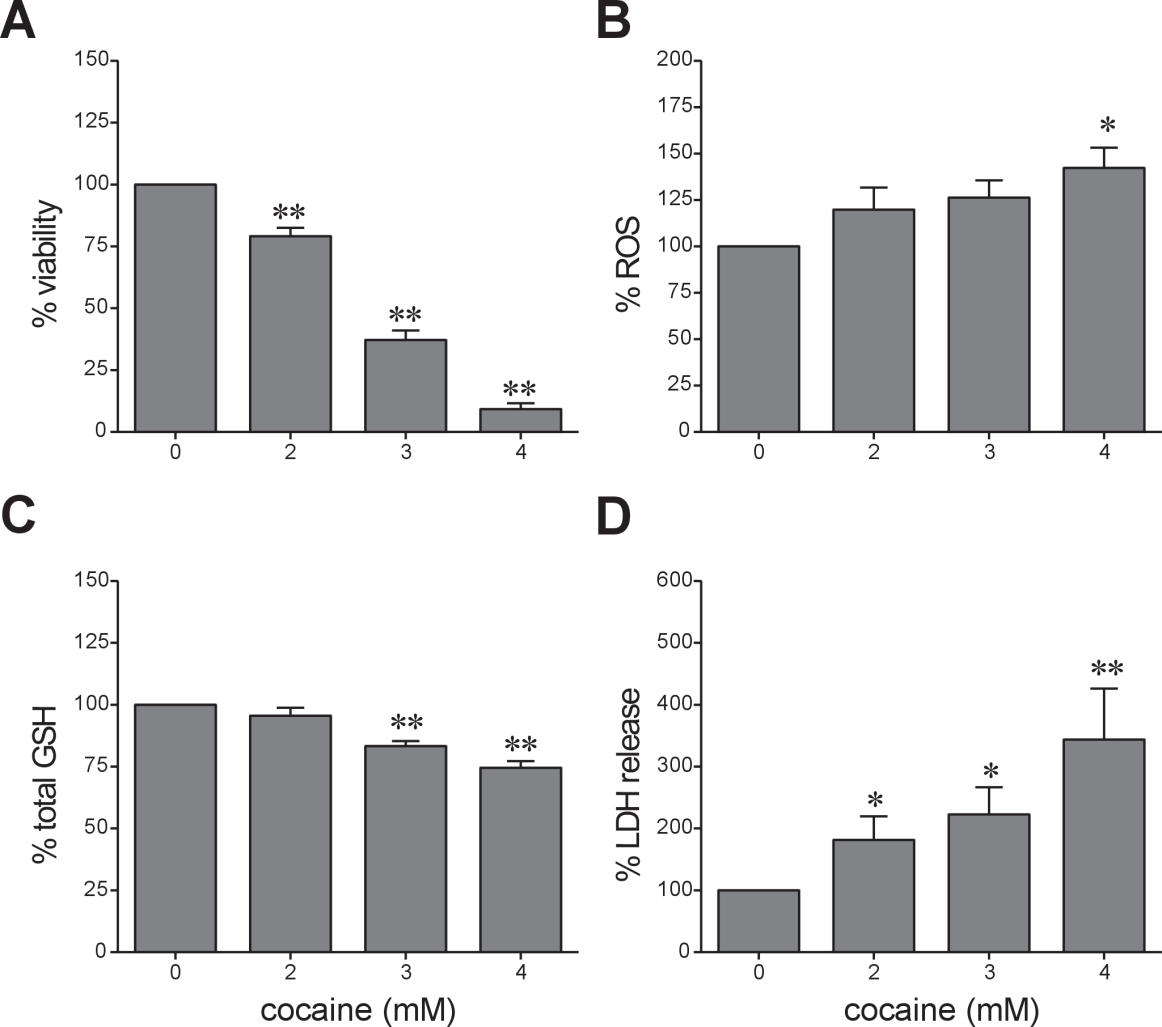
Measurement of the acute effects of cocaine exposure in astroglia-like cells on *cell viability* (A), *ROS production* (B), *total GSH levels* (C), and *LDH release* (D). Cells were treated with the indicated concentrations of cocaine for 1h. Cell viability was assessed using the crystal violet dye (0.1%) uptake protocol (*n* = 9). *ROS production* was assessed by loading cells with a H_2_DCFDA dye (10 μM, 30 min), followed by cocaine exposure in phenol red–free media. Dichlorodihydrofluorescein (DCF) was measured on a micro plate fluorometer with the excitation and emission filters set at 485 and 530 nm respectively (*n* = 4). Depletion in *GSH levels* were quantified on a plate reader following exposure to cocaine (*n* = 6). For measurements of *LDH release*, cells in phenol red–free media were treated with cocaine following which they were incubated in equal volumes of media and substrate from the assay kit (50 μl, 30 min) before being read on a micro plate reader (*n* = 12). Data for all measures are represented as mean ± S.E.M. (*n* = number of assays), *P<0.05; **P<0.001–0.01 w.r.t to control.

Acute exposure to cocaine caused a significant dose-dependent decrease in viability of astroglia-like cells at concentration greater than or equal to 2 mM (*n* = 9 assays, *F* = 210.8, *P*<0.01 at all concentrations with respect to control; Fig. 3A). The average cell viability (± S.E.M.) at 2, 3, and 4 mM cocaine was 79 ± 3.4, 37 ± 3.8 and 9 ± 2.4% respectively, compared with control (100%). The LC_50_ of cocaine in these cells was 2.7 mM. Study of cell viability by the MTS assay also yielded similar results with an LC_50_ of 2.8 mM cocaine (data not shown).

### ROS Production and Depletion of Total GSH Levels

Excess ROS production in cells could arise from cocaine–mitochondrial interaction resulting in its dysfunction [7, 8]. Indeed, cells treated with 2, 3 and 4 mM cocaine for 1h showed a dose-dependent increase in ROS release compared with control cells and at the highest concentration of cocaine tested (4 mM), there was as much as ~42% increase in ROS release (*n* = 4 assays; *F* = 3.5, *P*<0.05; Fig. 3B) suggesting that acute exposure to cocaine causes excessive release of ROS in astroglia-like cells. GSH is one of the most abundant antioxidants in cells. We found, concomitant with increases in ROS production, significant decreases in GSH levels at 3 mM cocaine or higher (*n* = 6 assays, *F* = 24.4, *P*<0.01; Fig. 3C). GSH levels were 17 ± 2.0 and 26 ± 2.7% of their control values (100%) at 3 and 4 mM cocaine, respectively.

### Plasma Membrane Damage and Disruption of F-Actin Filaments

**Figure 4:**
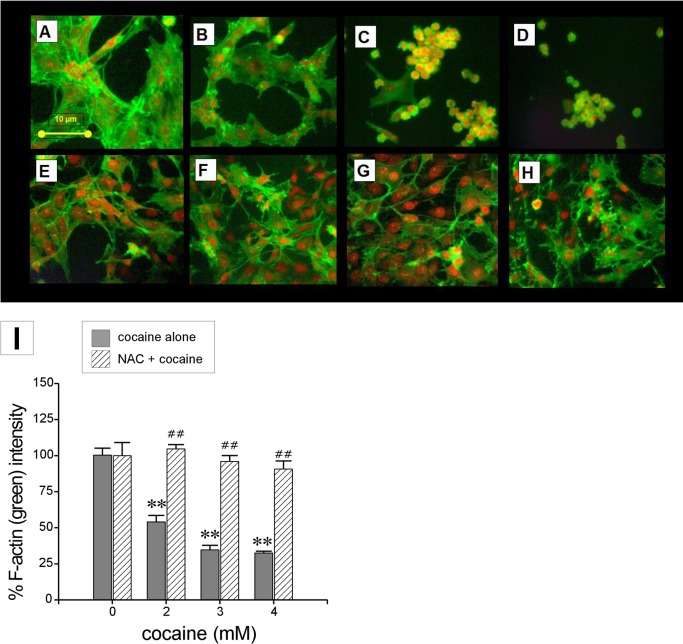
Effects of cocaine on nuclear morphology and F-actin cytoskeleton in astroglia-like cells. Photomicrographs showing cells treated with PBS (controls, **A**); cocaine alone 2, 3 and 4 mM, **B-D** respectively); NAC alone (5 mM, **E**); pretreatment with NAC (30 min) followed by a 1h exposure to cocaine (2, 3 and 4 mM, **F**-**H** respectively). Cells were incubated with PI (5 μg/ml) and then Alexa Fluor 488 Phalloidin (6.6 μM) before being imaged on an inverted fluorescent microscope with a 40x objective. Images show changes in structural cytoskeleton captured by F-actin phalloidin staining and counterstaining with PI. Fluorescence intensity for phalloidin staining (**I**) was quantified using image J software. Data represent mean intensity ± S.E.M. (*n* = 3, **P<0.01 significance of comparison with corresponding control; ^##^ *P*>0.05 insignificance of comparison with corresponding control.

LDH is a ubiquitous constituent of cell cytoplasm. Its presence in the extracellular supernatant is indicative of a compromised plasma membrane following an insult. We observed a significant dose-dependent release of LDH from astroglia-like cells in response to acute exposure to cocaine (*n* = 12 assays, *F* = 4.0, *P*<0.05 at all concentrations with respect to control; Fig. 3D). LDH release was increased on average by 81 ± 39 and 123 ± 44% of their control values at 2 and 3 mM respectively and at 4 mM cocaine, there was a greater than 3-fold increase (344 ± 83%) in the amount of LDH. These data suggest that acute exposure to cocaine damages astroglia-like cell membrane. Yet, despite its effect on plasma membrane, cocaine did not induce nuclear or chromatin fragmentation (karyolysis) as evidenced by PI (red) staining. However, a significant dose-dependent (*n* = 3, *F* = 28.4, *P*<0.01) decrease in phalloidin staining (Fig. 4I) suggests that acute cocaine exposure may disrupt F-actin cytoskeleton (green) in astroglia-like cells (Fig. 4A-D). The average decreases in phallodin staining compared to control were 54 ± 5, 35 ± 3, 33 ± 1% at 2, 3 and 4 mM cocaine, respectively (Fig. 4I). These data implicate the cytoskeleton as one of the primary cellular targets of injury arising from acute exposure to cocaine.

### NAC Pretreatment Prevents Cocaine-Induced Morphological Changes

**Figure 5:**
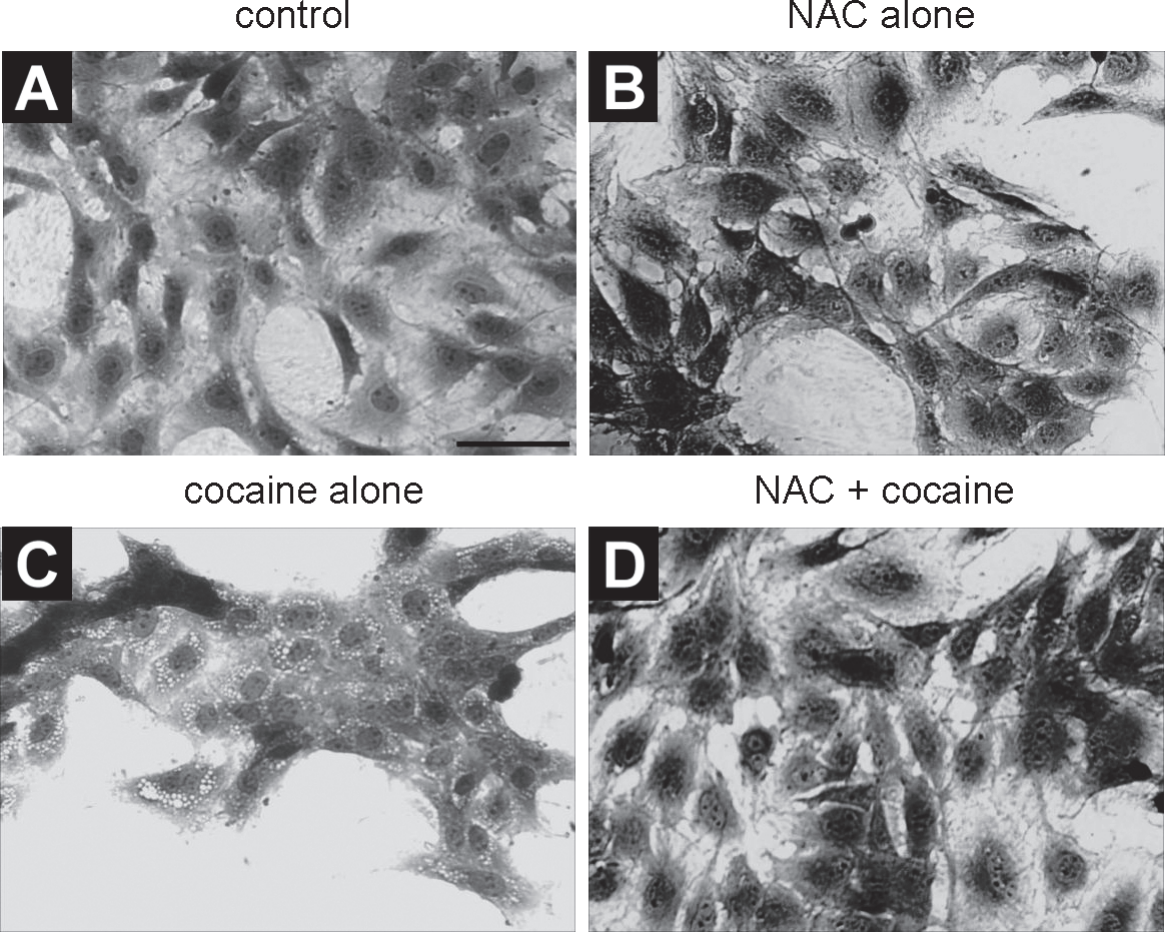
Cocaine-induced morphological alterations are ameliorated with NAC pretreatment. Cells were treated with PBS (control, 1h) (**A**); NAC alone (5 mM, 30 min) (**B**); cocaine alone (2 mM, 1h) (**C**); pretreated with NAC (5 mM, 30 min) followed by exposure to cocaine (4 mM, 1h) (**D**). Cells are stained with crystal violet and photographed under an inverted phase contrast 1X-70 Olympus microscope with 40x objective. Scale bar: 50 μm.

Microscopic observation of crystal violet stained cells revealed that compared with untreated cells (Fig. 5A), there were no significant morphological differences in either the NAC pretreated control cells (Fig. 4B) or NAC pretreated cells exposed to cocaine (4 mM) (Fig. 5D). A noteworthy difference was the conspicuous absence of vacuoles in the NAC pre-treated cells that were exposed to cocaine compared with those that were exposed to cocaine alone (Fig. 5C, D). We verified that NAC by itself or in combination with cocaine did not cause LDH release nor did it disrupt F-actin cytoskeleton in cells (Fig. 4E). We found almost equal levels of phallodin in NAC control cells and cells co-treated with cocaine (*P*>0.05; Fig. 4I).

### Attenuation of Cocaine Toxicity with NAC Pretreatment

**Figure 6:**
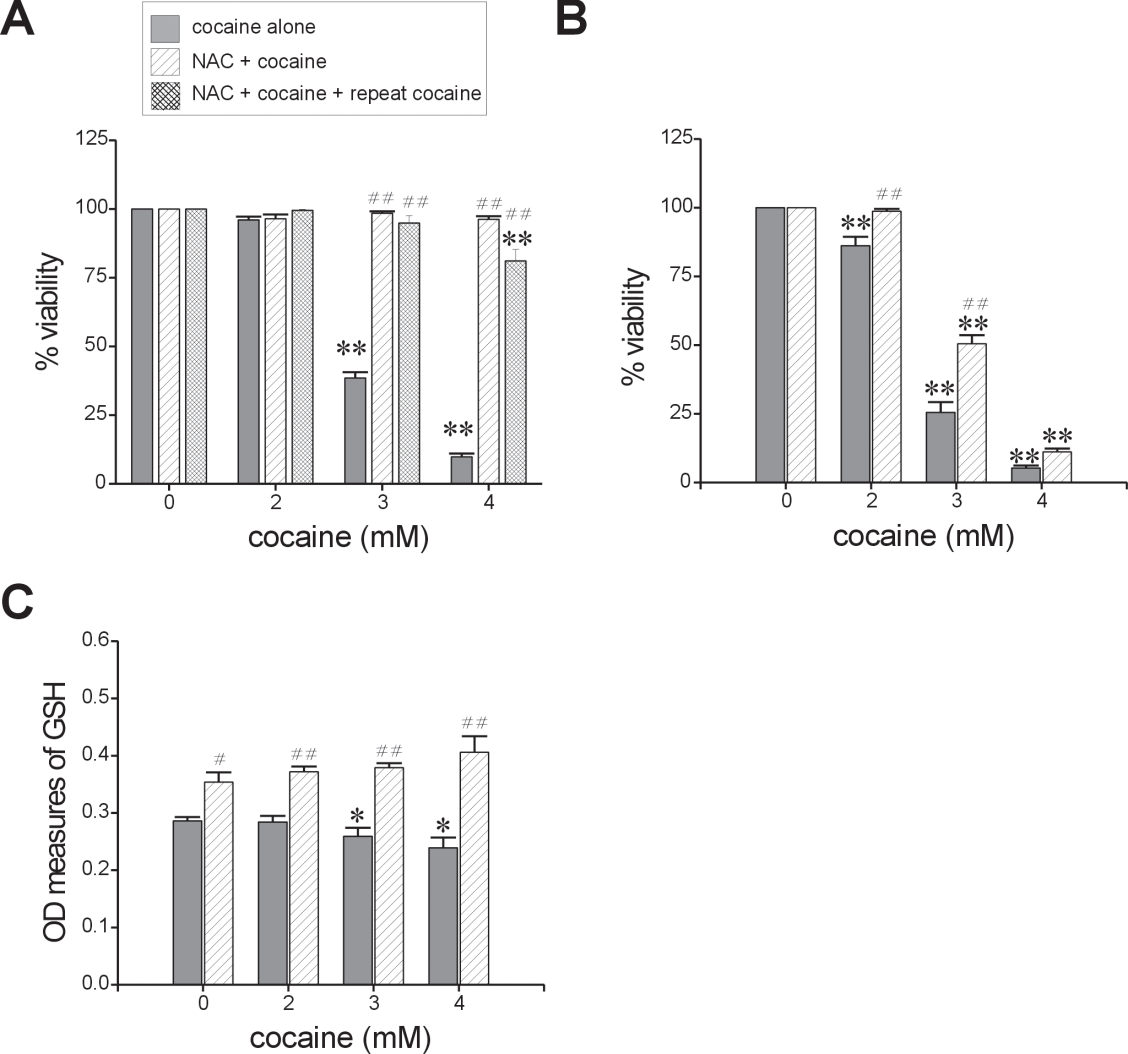
NAC mitigates cocaine-induced toxicity. Measurements of cell viability with NAC pretreatment (*n* = 12, **A**) or post NAC exposure (*n* = 16, **B**) and total GSH levels (*n* = 8–12, **C**) with NAC pretreatment (5 mM, 30 min) followed by exposure to the indicated concentrations of cocaine for 1h. Data represent mean ± S.E.M. (*n* = number of assays), *P<0.05, **P<0.01–0.001 significance of comparison with corresponding control; ^##^ *P*<0.001 significance of comparison between cocaine alone and cocaine + NAC.

A dose-dependent viability assay indicated that NAC alone (1–5 mM, 1h) was not toxic to astroglia-like cells [21]. Even at 5 mM NAC, treated cells were as healthy and viable as untreated ones (data not shown) prompting the use of two concentrations of NAC (2.5 and 5 mM) in assaying its protective role against cocaine-induced toxicity. Cells were pretreated with 2.5 or 5 mM NAC for 30 min before exposure to cocaine (2–4 mM) for 1h. The viability data revealed that while NAC pretreatment at 2.5 mM rendered 60–70% cell protection against cocaine-induced toxicity (data not shown), 5 mM NAC could provide full protection (100%, *n* = 12 assays, *F* = 824, *P*<0.01; Fig. 6A) and was therefore chosen as the preferred dose for the remainder of this study. In an attempt to see if NAC pretreatment could decrease cocaine toxicity when cells are repeatedly exposed to cocaine, the media of cells treated with NAC and cocaine were exchanged for fresh media, before cocaine retreatment (2–4 mM, 1h). Our data (Fig. 6A) suggests that cells were viable despite repeated cocaine treatments (viability 81–100%; *n* = 12 assays, *F* = 288, *P*<0.01) in contrast with cells that were treated with cocaine alone. Interestingly, post NAC exposure caused a marginal but significant (*n* = 16 assays, *F* = 365, *P*<0.01) increase in cell viability compared to cells treated with cocaine alone (Fig. 6B). The average increase in the viability due to post NAC exposure was 13, 25 and 6%, respectively to 2, 3 and 4 mM cocaine exposure. The LC_50_ was increased from 2.7 to 3 mM cocaine.

### NAC Pretreatment Increases Intracellular GSH Levels

GSH is a tripeptide, synthesized from L-cysteine, L-glutamic acid and glycine. NAC enters the cells easily and releases cysteine upon hydrolysis. The released cysteine can serve as a precursor for GSH biosynthesis in astrocytes. In order to determine if NAC pretreatment alters GSH levels in the presence of cocaine, data were analyzed under absorbance at 412 nm (Fig. 6C). GSH levels in NAC pretreated control cells were significantly higher compared with naïve control cells (no cocaine). This observation supports the hypothesis that cells did indeed utilize the cysteine residue from NAC to synthesize glutathione during the pretreatment period. Exposure to cocaine further increased GSH levels at all concentrations tested, and these levels were significantly greater than those in cocaine exposed cells that were not pretreated with NAC (*n* = 8–12 assays, *F* = 16.3, *P*<0.01).

### Attenuation of Histone Methylation by NAC

**Figure 7:**
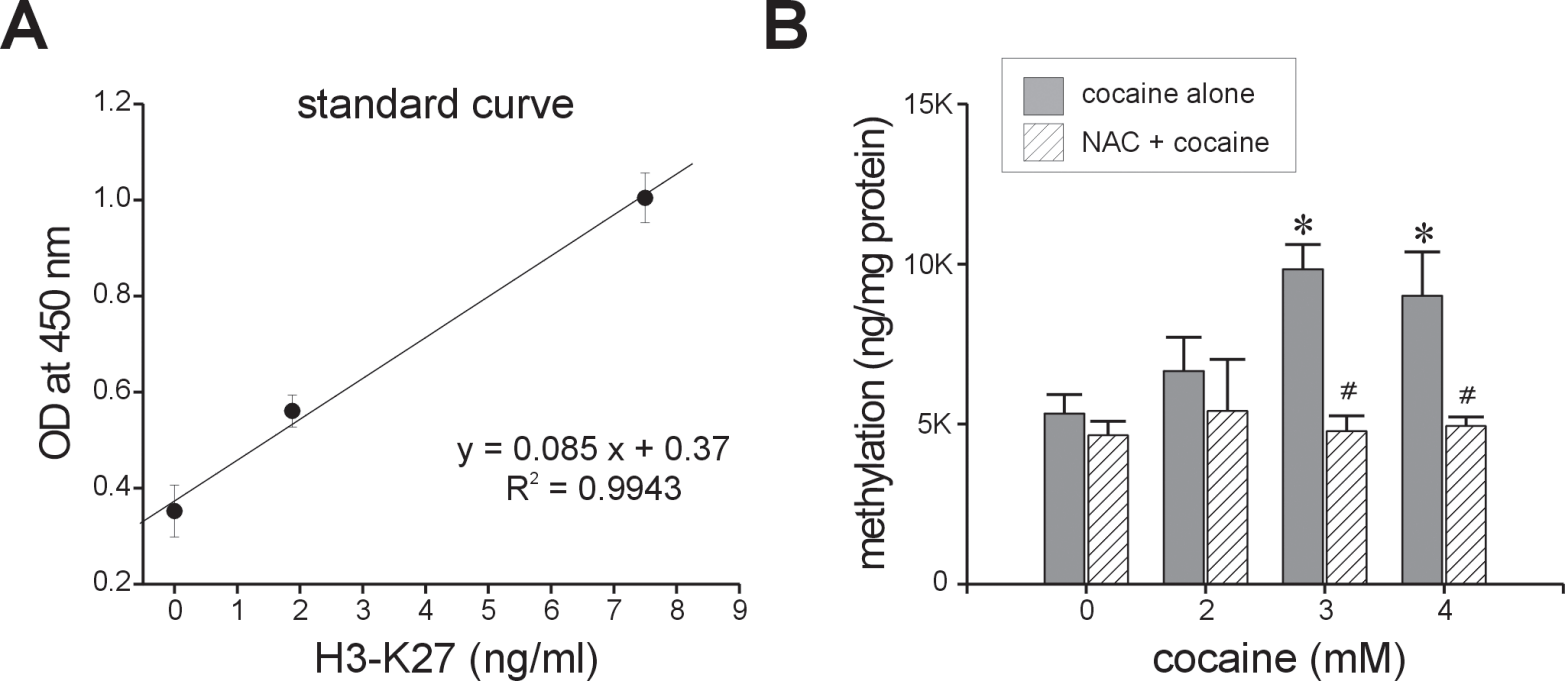
Histone methylation with acute cocaine treatment. Standard curve of methylated H3-K27 (**A**). Cells were pretreated with NAC (5 mM, 30 min; n = 6) followed by exposure to the indicated concentrations of cocaine for 1h (**B**). Data represent mean ± S.E.M. (*n* = number of assays), *P<0.05, significance of comparison with corresponding control; ^#^ *P*<0.05, significance of comparison between cocaine alone and cocaine + NAC.

Histone methylation, a form of epigenetic chromatin remodeling, may have a major role in drug addiction. Although this process can occur in any of the histones H2, H3 or H4, methylation of histone H3 at K (lysine) 27 position in particular has been associated with gene repression [39] influencing genome function at large and linked to depression-like behavior in animals [40]. To investigate if cocaine treatment could methylate histone H3 at K27 in astroglia-like cells, we used the standard curve for methylated H3-K27 (slope: 0.085; Fig. 7A) to quantify the extent of methylation in our cultures. We found that the amount of methylation at K27 increased significantly as a function of increasing concentrations of cocaine (*n* = 6 assays, *F* = 4.6, *P*<0.05 at 3 and 4 mM cocaine; Fig. 7B). For instance, compared with control cells (0%), the increase in methylation observed with 3 and 4 mM cocaine was 85 ± 15% and 69 ± 26%, respectively. Interestingly, while pretreatment of naïve control cells with NAC (5 mM, 30 min) did not alter base line methylation, exposure of these cells to cocaine now abrogated its effects on methylation. Thus, in the presence of NAC, histone methylation levels in cocaine-exposed cells were comparable to those of control cells (no cocaine, Fig. 7B). These data clearly indicate that NAC pretreatment inhibits cocaine-induce methylation of histones in astroglia-like cells.

## Discussion

According to the National Institute of Drug Abuse [41], ~3.6 million Americans abuse cocaine on a regular basis, many at an early age, exasperating the problem of cocaine dependency–a major cause of drug-related deaths within the US [42]. Cocaine is highly lipophilic and reaches various domains of the brain easily. Astrocytes, part of the frontline defense against chemical damage in the brain [43], are potential targets of substances of drug abuse. Studies have demonstrated that drugs of abuse like cocaine cause toxic effects in astrocytes by altering their morphology and cell size *in vivo* [44–46]. Cocaine entry into astrocytes could trigger several early response changes which adversely affect their survival. Despite their importance, no studies to date have looked at these acute changes in astrocytes upon cocaine exposure or determined the minimal doses of cocaine needed to compromise their viability *in vitro*. Because *C6* astroglia-like cells exhibit several similarities to astrocytes in terms of gene expression [15], presence of enzymes [16] and GFAP expression (Fig. 1; [29]), we used them to identify the early response changes following acute cocaine exposure.

### Cocaine Concentrations

Cocaine concentrations needed to elicit pharmacological responses in brain cells from human addicts have ranged from nano- to micromolar [47] and under *in vitro* conditions have extended well into the millimolar range [7, 48–51]. Hence, the use of even 10 mM cocaine–2 to 5 times the concentrations used in this study–at even longer incubation intervals are not atypical [48, 50, 52] despite cocaine's relatively short half-life of ~1 hour *in vivo* [31, 32]. Furthermore, cocaine doses 1 mM or less, fail to show significant death of astroglia-like cells under *in vitro* conditions (data not shown), an observation consistent with previous reports [49].

The apparent discrepancy between measurement of cocaine levels *in vivo* and those required to elicit a cellular response *in vitro* may be attributed to, among other factors, thermodynamics, wherein the latter offers better control of environmental conditions and therefore greater accuracy of measurements. Nonetheless, the difficulty of correlating *in vivo* effects based on *in vitro* data needs to be clearly acknowledged. Likewise, measurements of cocaine levels *in vivo* also do not necessarily reflect actual amounts of cocaine consumed by an addict [48] owing to, for instance, the development of drug tolerance [53] in which a well-adapted abuser can take in as much as 5g of cocaine per day. Hence, an accurate assessment cocaine levels in the brain requires factoring in of issues such as drug tolerance and/or frequency of use [54] and/or its hydrolysis by blood esterases [55]. The cocaine concentrations used in the present study–deemed of physiological relevance–are not only based on LC_50_ and EC_50_ values found in the literature [50,51, 56, 57], but our own assessments of the minimal concentrations needed to induce detectable changes following an acute exposure [7, 8]. The present study establishes that the minimal concentrations of cocaine required to compromise astroglia-like cell viability significantly is 2 mM under *in vitro* conditions of acute exposure lasting 1h. On average, this translates to ingestion of ~3.3 g of cocaine by an adult human (assuming a total blood volume of ~5 L) and/or ~5 mg of cocaine by a juvenile-adult rat (120 g, assuming a blood volume of 64 ml/Kg), values that are well within the realm of possibility / experimental research. In addition, we have confirmed that the observed pathology in astroglia-like cells following acute exposure to cocaine is recapitulated in both neuronal and non-neuronal tissue [58–60] and under other conditions of oxidative stress including methamphetamine neurotoxicity [61].

### Early Response Changes

**Figure 8:**
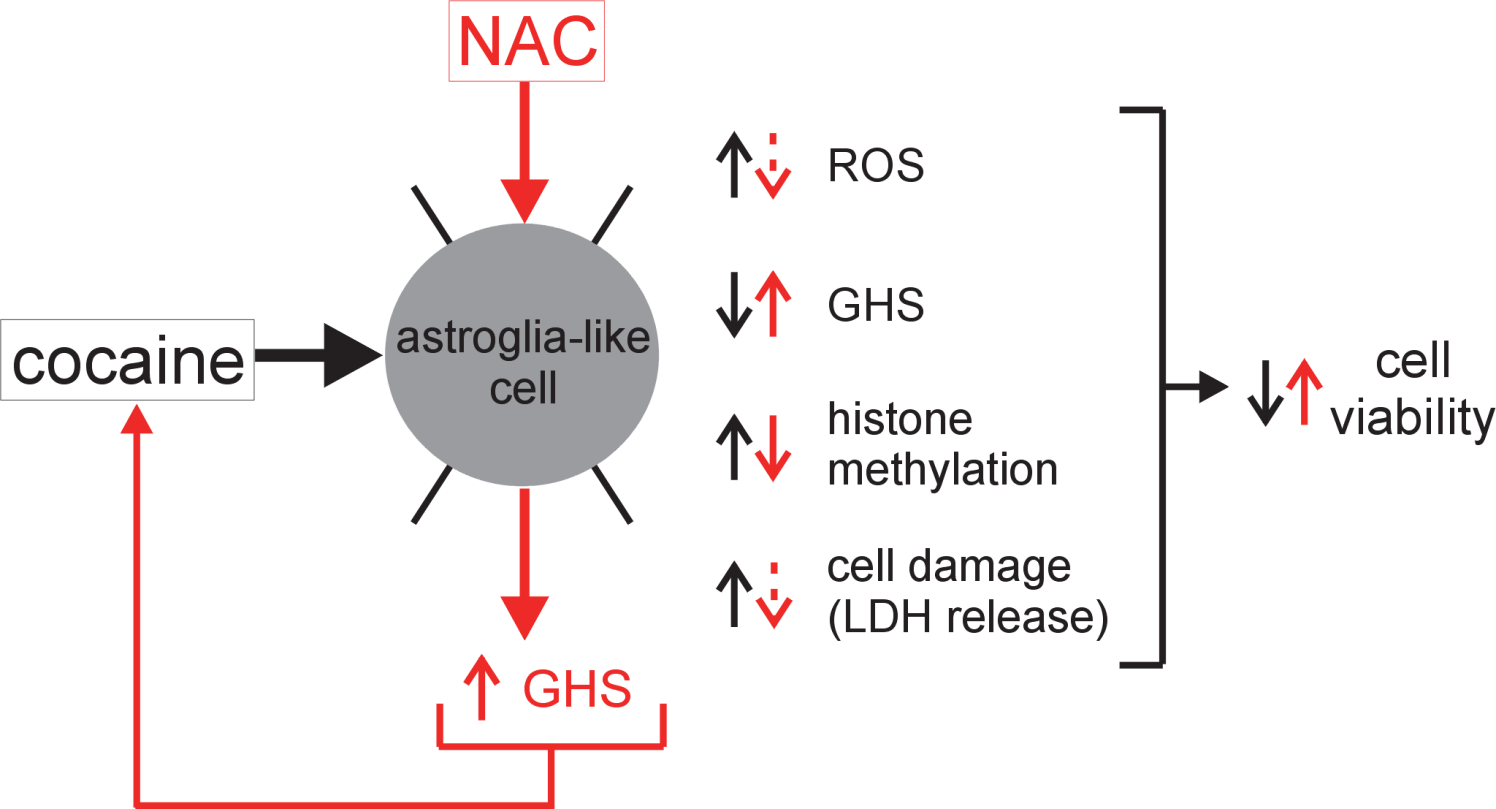
Schematic representation of NAC protection against cocaine toxicity. The acute effects of cocaine (*black*) on astroglia-like cells and the mode of protection rendered by NAC (*red*) through measured (*solid*) and/or inferred (*broken*) increases (*up arrows*) or decreases (*down arrows*) in levels of the indicated parameters.

Drugs that inhibit mitochondrial enzymes interfere with cellular respiration often resulting in the production of excess ROS [62]. GSH, a major antioxidant in cells, counteracts the increased ROS levels to provide protection. We observed that cocaine treatment not only increased ROS production in astroglia-like cells (Fig. 3B), but concomitantly decreased GSH levels–a double insult (Fig. 3C). While increased ROS levels confirm that cocaine interferes with mitochondrial energy metabolism, an observation consistent with our previous studies [7, 8], decreases in astrocytic GSH levels have also been reported in the literature [63]. Depletion in GSH levels make cells more vulnerable to oxidative injury which could led to cell death via cytoskeleton and plasma membrane damages. Cocaine-induced cytoskeletal damage was reported in neurons [64], but there are no studies of cocaine's effects on astrocytic cytoskeleton. This is the first report to show rapid damage to F-actin filaments of cytoskeleton in astroglia-like cells upon cocaine exposure (Fig. 4). The dose-dependent LDH release (Fig. 3D) further supports the view that cocaine-induced ROS acts at the level of cytoskeleton and membrane to cause cell death (Fig. 3A). The presence of intact nuclei as indicated by PI staining (Fig. 4) suggests that cocaine-induced cell death is not mediated through apoptosis. Rather, our data collectively suggest that the mechanism of cytotoxicity in *C6* astroglia-like cells is through the early response changes (Fig. 8). Pharmacological interventions that inhibit these early response changes of cocaine-induced toxicity in astrocytes may not only prevent astrocytic cell death but neuronal cell death as well owing to their interdependency [12, 13].

### NAC Offers Protection

Astrocytes contain higher levels of GSH compared to other cell types in the brain [65]. GSH provides both extracellular protection and serves as a precursor for intra neuronal GSH synthesis. Thus, depletion of GSH levels in astrocytes not only makes them prone to oxidative injury but also causes imbalances in the redox potential of neighboring neurons. In our study, given that early response changes underlie loss of astroglia-like cells upon acute exposure to cocaine, we sought ways to counteract and/or prevent them to boost survival rates. Based on the observation that some early response changes such as increased ROS and decreased GSH levels (Figs. 3B,C) could contribute to the cell death, we reasoned that pretreatment with NAC–a well-known antioxidant and therapeutic agent for several oxidant-related CNS diseases [66]–could surmount cocaine toxicity in astroglia-like cells. Recently this compound has been viewed as a potential pharmacological drug for treating cocaine dependence [67].

We observed that NAC pretreatment clearly enabled astroglia-like cells to retain their morphology upon cocaine exposure (Fig. 5) while maintaining 100% viability (Fig. 6A). Astroglia-like cells pretreated with NAC showed no signs of vacuolization even at high levels of GSH (Fig. 5B, D). Interestingly, NAC pretreated cells when subjected to repeated cocaine exposure, maintained a high cell viability (81–100%), suggesting that NAC could provide protection against recurring drug abuse in addicts. Further research, however, is warranted to ascertain if this result is also applicable to cases in which cocaine addicts have already sought medical treatment following a prolonged exposure. The results of our study mimicking this situation clearly indicate that post NAC exposure significantly rescues cells from cocaine toxicity, albeit to a lesser degree (6–25%, Fig. 6B)– a novel observation.

The NAC-mediated protection did not appear to result from NAC-cocaine complex formation because GSH levels were significantly elevated upon cocaine exposure (Figs. 6C, 8) and may have played a major protective role against cocaine toxicity. Cell viability and GSH production were also unaffected when NAC was withdrawn following pretreatment [21] and, as can be seen from the present study, GSH levels remained invariant (Fig. 6C) between NAC pretreated control cells (no cocaine) and NAC pretreated cells that were exposed to 2–4 mM cocaine. These data suggest that GSH is most likely synthesized during the pretreatment period to render protection during subsequent exposure of the cells to cocaine. NAC's protection also appears to extend to cocaine-induced methylation in astroglia-like cells as the increased methylation at H3-K27 observed in this study (Fig. 7B) was abrogated by pretreatment with NAC. Studies have shown that histone methylation is associated with neurodegenerative diseases such as Parkinson's, schizophrenia, Alzheimer's disease, and other cognitive defects [68] and increased methylation may put cocaine addicts at a risk for these diseases early in life.

## Conclusion

We identified several early response changes such as alterations in mitochondrial membrane potential [7], mitochondrial respiratory status [8], vacuolation (Fig. 2B), ROS production (Fig. 3B), cellular GSH levels (Fig. 3C), F-actin cytoskeleton (Fig. 4), and histone methylation (Fig. 7B) in *C6* astroglia-like cells following acute exposure to cocaine. Our data provide compelling evidence to support the hypothesis that inhibition of the early response changes by NAC enhances cell survival through increased GSH levels. Compounds which support GSH synthesis could therefore mitigate toxicity of early response events in cells exposed to cocaine [22]. The recapitulation of cocaine-induced changes observed in our model (e.g. vacuolization [58]) lends credibility to its use for studying cocaine induced toxicity *in vivo*.
